# Bipedicled DIEP Flap to Reduce Complications in Patients Treated with Radiotherapy: A Single-Center Case Series

**DOI:** 10.3390/jcm15218245

**Published:** 2026-10-23

**Authors:** Federico Ziani, Corrado Rubino, Marco Borrini, Sebastiano Emanuele Palladino, Sofia De Riso, Paola Pentangelo, Emilio Trignano

**Affiliations:** 1 Department of Medicine, Surgery and Pharmacy, University of Sassari, 07100 Sassari, Italy; zianifederico@gmail.com (F.Z.); corubino@uniss.it (C.R.); s.palladino@studenti.uniss.it (S.E.P.); sofia.deriso@aouss.it (S.D.R.); etrignano@uniss.it (E.T.); 2 Plastic Surgery Unit, University Hospital Trust of Sassari, 07100 Sassari, Italy; 3 Plastic Surgery Unit, Department of Medicine, Surgery and Dentistry, University of Salerno, 84081 Baronissi, Italy; ppentangelo@unisa.it

**Keywords:** Breast reconstruction, microsurgery, free flaps, breast cancer, radiotherapy, DIEP flap, bipedicled DIEP flap

## Abstract

**Background:** Autologous breast reconstruction after chest-wall radiotherapy (RT) remains challenging because radiation-induced vascular injury may impair flap perfusion and wound healing. Bipedicled deep inferior epigastric perforator (DIEP) flaps anastomosed to two separate recipient systems may provide greater vascular redundancy in this setting. **Methods:** This retrospective single-center case series, reported according to the PROCESS 2025 guideline, included 26 consecutive women who underwent unilateral bipedicled DIEP flap breast reconstruction in the setting of chest-wall RT between September 2024 and March 2026, with a minimum follow-up of 6 months. Outcomes were total flap loss, flap-related complications, donor-site morbidity, and BREAST-Q scores. **Results:** One pedicle was anastomosed to the internal mammary vessels and the other to an axillary branch, most often the circumflex scapular artery (18/26; 69.2%). Mean operative time was 7 h 9 min ± 47 min. No total flap loss occurred. One arterial complication (3.8%) involved the internal mammary pedicle, and the flap survived. Abdominal bulge occurred in 5 patients (19.2%). Mean BREAST-Q scores were ≥90 in all domains. **Conclusions:** In this case series, bipedicled DIEP flap reconstruction was associated with no total flap loss after chest-wall RT, without an apparent increase in early donor-site morbidity. The biological condition of the recipient bed may deserve consideration when selecting flap configuration; larger prospective studies are required to confirm these findings.

## 1. Introduction

Breast cancer is the most commonly diagnosed cancer among women worldwide [1], and mastectomy remains one of the main surgical treatments. After mastectomy, an increasing number of patients choose breast reconstruction to improve body image and quality of life [2]. Two main reconstructive options are currently available: implant-based reconstruction, using tissue expanders and breast implants, and autologous tissue reconstruction [3].

Although implant-based reconstruction is the most performed technique worldwide [4], accounting for approximately 80% of post-mastectomy reconstructions, autologous reconstruction is often considered the gold standard for selected patients because it provides more natural and long-lasting results. In addition, it is associated with a lower risk of long-term complications such as implant rupture, capsular contracture, and secondary infection.

Among autologous techniques, the deep inferior epigastric perforator (DIEP) flap has become one of the preferred options. It allows the transfer of well-vascularized abdominal tissue while preserving the abdominal muscles, reducing donor-site morbidity [5]. However, the best approach to breast reconstruction in patients requiring post-mastectomy radiotherapy (PMRT) is still debated. PMRT can negatively affect reconstructive outcomes, especially in implant-based reconstruction, making surgical planning more challenging [6].

In this study, we investigated the outcomes of autologous breast reconstruction with DIEP flaps in patients who had previously received PMRT. Because reconstruction in irradiated patients remains challenging, several surgical strategies have been proposed to improve both functional and aesthetic results while reducing complications [7]. We present our experience with a series of irradiated patients who underwent DIEP flap breast reconstruction,. Bipedicled DIEP flaps may provide better perfusion, improved volume distribution, and greater reliability in compromised recipient sites [8,9]. However, these potential advantages must be balanced against the increased technical complexity of the procedure and the possibility of higher donor-site morbidity [10,11,12].

## 2. Material and Methods

### 2.1. Study Design and Patients

This is a retrospective, single-center case series of 26 consecutive female patients who underwent unilateral breast reconstruction with a conjoined bipedicled DIEP flap in the setting of chest-wall radiotherapy (RT) at the Plastic Surgery Unit of the University Hospital Trust of Sassari between September 2024 and March 2026. Data were retrospectively collected from a prospectively maintained institutional database. During the study period, the bipedicled configuration was the default for all irradiated patients undergoing DIEP flap reconstruction at our institution. The case series is reported in line with the PROCESS 2025 guideline [13].

One perforator was included per pedicle, for a total of two perforators per flap. The transferred tissue corresponded to Hartrampf zones I and II; zones III and IV were trimmed during flap shaping.

Inclusion criteria were (i) previous mastectomy; (ii) chest-wall RT in one of the following settings: neoadjuvant RT; delayed salvage RT, not initially planned and administered after a prior implant-based reconstruction; or planned RT, administered after mastectomy in patients reconstructed immediately with a DIEP flap with a known preoperative indication for RT; (iii) unilateral reconstruction with a bipedicled DIEP flap; and (iv) a minimum follow-up of 6 months. Exclusion criteria were bilateral reconstruction, combined use of additional flaps, history of major abdominal surgery, and loss to follow-up before 6 months.

**Figure 1:**
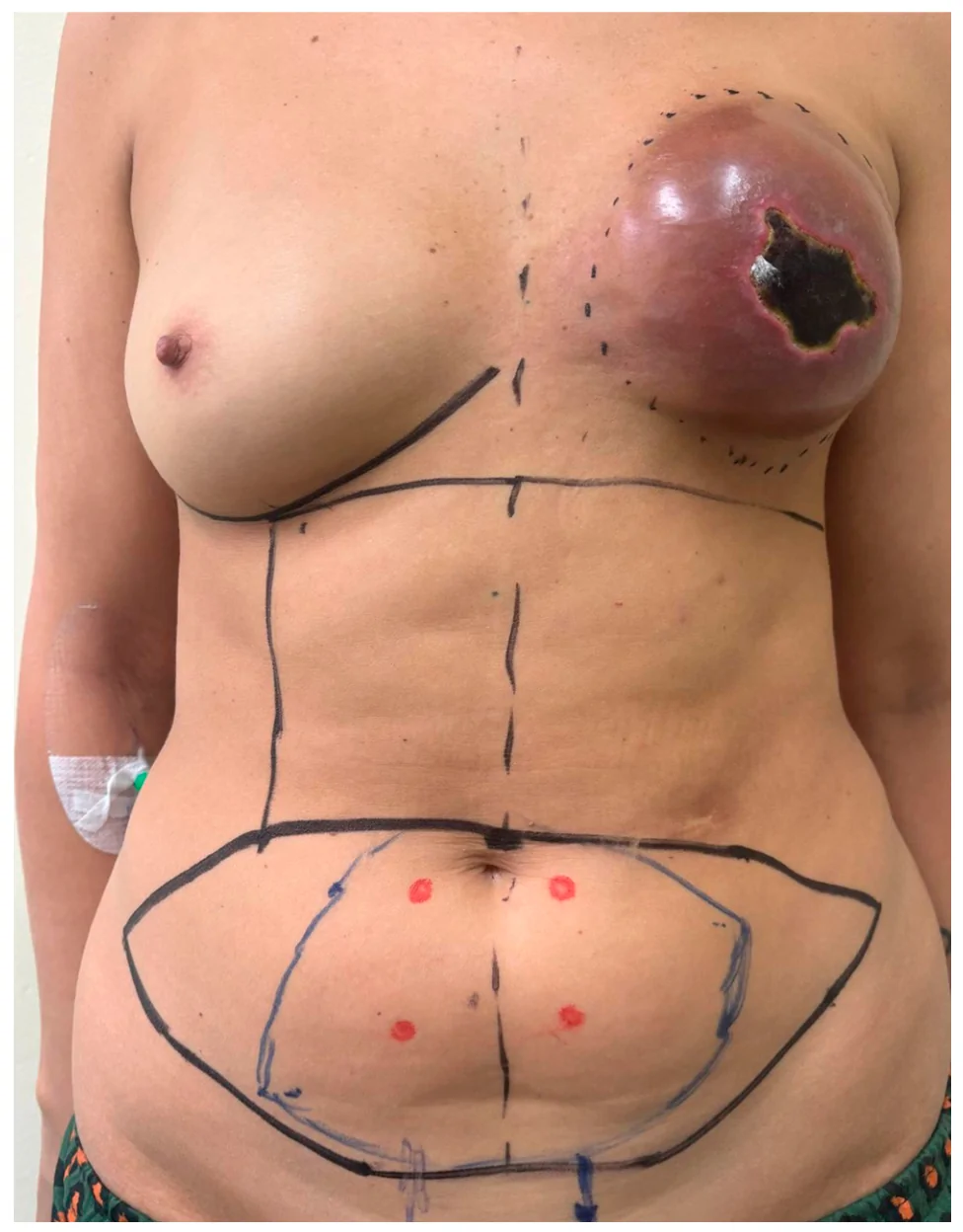
Preoperative markings in a patient with a previously irradiated left breast and a failed implant-based reconstruction complicated by skin necrosis. The abdominal flap is outlined in black, and the portion planned for transfer in blue; the perforators identified with handheld Doppler are marked with red dots, and the midline and inframammary folds are also marked. Dash circle indicate area damaged by radiotherapy.

Before DIEP flap elevation, perforators were marked out with a handheld Doppler (handheld ecodoppler huntleigh, hoffman estates, Illinois) (Figure 1). All procedures were performed by the same senior surgeon, who performed both flap elevation and microvascular anastomosis, according to a standardized two-team surgical technique explained in the following section.

### 2.2. Data Collection

The following variables were collected: age, weight, body mass index (BMI), recipient vessels used for each pedicle, operative time (hours), and length of follow-up.

### 2.3. Outcomes

The primary outcome was total flap loss. Secondary outcomes were vascular complications, skin necrosis, fat necrosis, minor recipient-site complications (a composite of seroma, hematoma, infection, and wound dehiscence with delayed healing), and donor-site morbidity (abdominal bulge and abdominal wound dehiscence). Patient-reported outcomes were assessed with the BREAST-Q at the 6-month follow-up visit. Because no total flap loss occurred, all patients were eligible for BREAST-Q assessment.

### 2.4. Ethics

The case series was conducted in accordance with the Declaration of Helsinki. Owing to the retrospective design of the study and the use of fully anonymized data, formal ethical review and approval, as well as specific informed consent for study participation, were waived in accordance with local institutional guidelines.

### 2.5. Surgical Technique

Our equipe works under a standardized double-equipe protocol to optimize operative time and avoid excessive duration. Standardization is essential to ensure proper coordination between teams.

**Figure 2:**
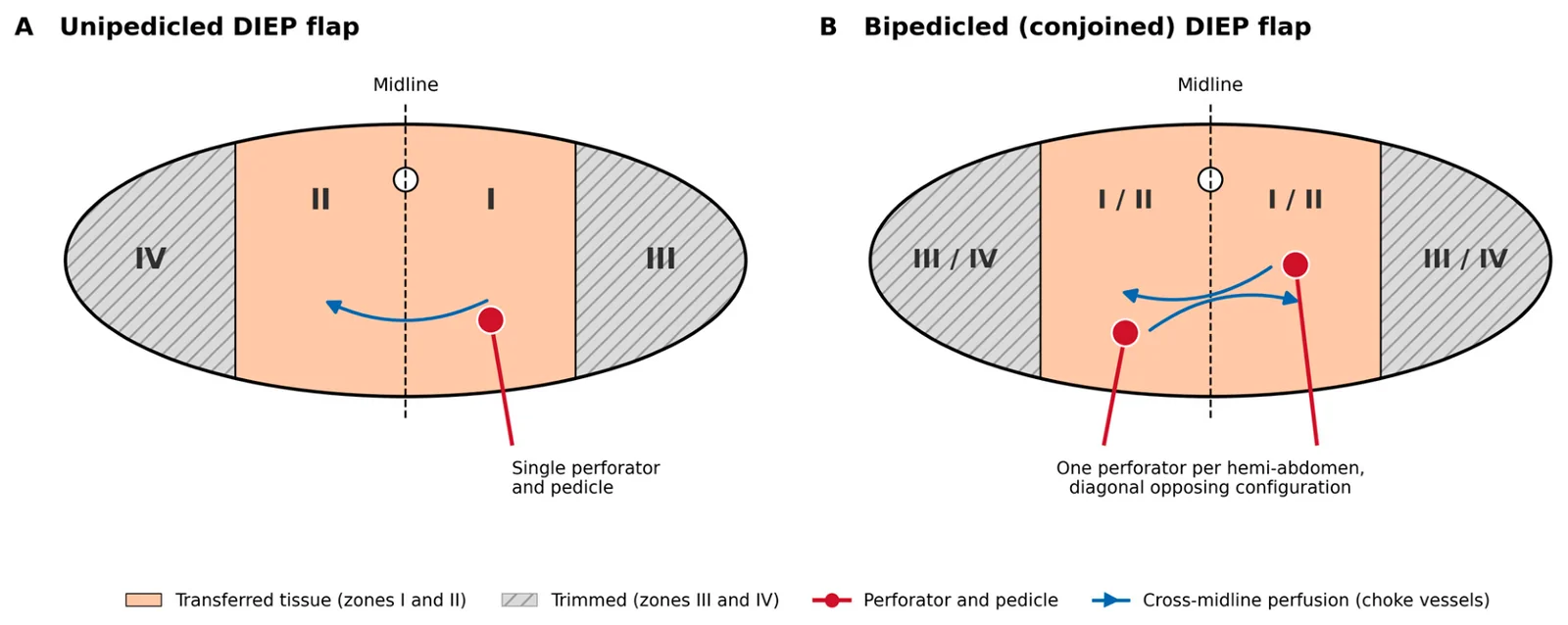
Schematic representation of flap design according to Hartrampf’s perfusion zones: (**A**) Unipedicled DIEP flap based on a single perforator: Zone I overlies the pedicle, Zone II lies across the midline, and Zones III and IV lie laterally. (**B**) Conjoined bipedicled DIEP flap based on one perforator per hemi-abdomen, preferably in a diagonal opposing configuration: each pedicle directly perfuses its own hemi-flap and the contralateral hemi-flap through choke vessels across the midline (arrows), so that the flap can survive on either pedicle. In both configurations, Zones I and II are transferred, and Zones III and IV are trimmed.

Our technique is based on Hartrampf’s zones of perfusion (Figure 2): since each pedicle also provides perfusion to the contralateral zone, we perform a bipedicled flap based on two contralateral perforators so that even if one pedicle were to undergo a complication, the contralateral one ensures flap survival. Whenever possible, we select perforators in a diagonal opposing configuration to optimize blood perfusion [14].

Only conjoined bipedicled DIEP flaps were used in this series; no reconstructions combining two separate flaps were performed. Because the flap is raised as a single unit without division at the midline, dissection is performed sequentially on each hemi-abdomen (see phases below) so that each perforator and its pedicle can be dissected with adequate exposure. Perforators are selected on the basis of preoperative handheld Doppler mapping, choosing one perforator per side, preferably in a diagonal opposing configuration. The use of two distinct recipient systems, described below, allows each pedicle to reach its recipient vessels without crowding or kinking during flap inset, and flap orientation on the chest wall is planned accordingly.

Our protocol divides the surgery into three phases.

In the first phase, the first equipe proceeds to elevate the DIEP flap on the side contralateral to the mastectomy, while the second equipe prepares the vessels of the receiving site: branches of the axillary vessels (circumflex scapula artery > thoracodorsal artery > lateral thoracic artery) and a perforator of the internal mammary (we also remove the third rib for better handiness and flap inset). If the mastectomy must be performed at the same time, mastectomy is performed by breast unit surgeons before the vessel preparation, while the first equipe proceeds to prepare the vessel as described above.

**Figure 3:**
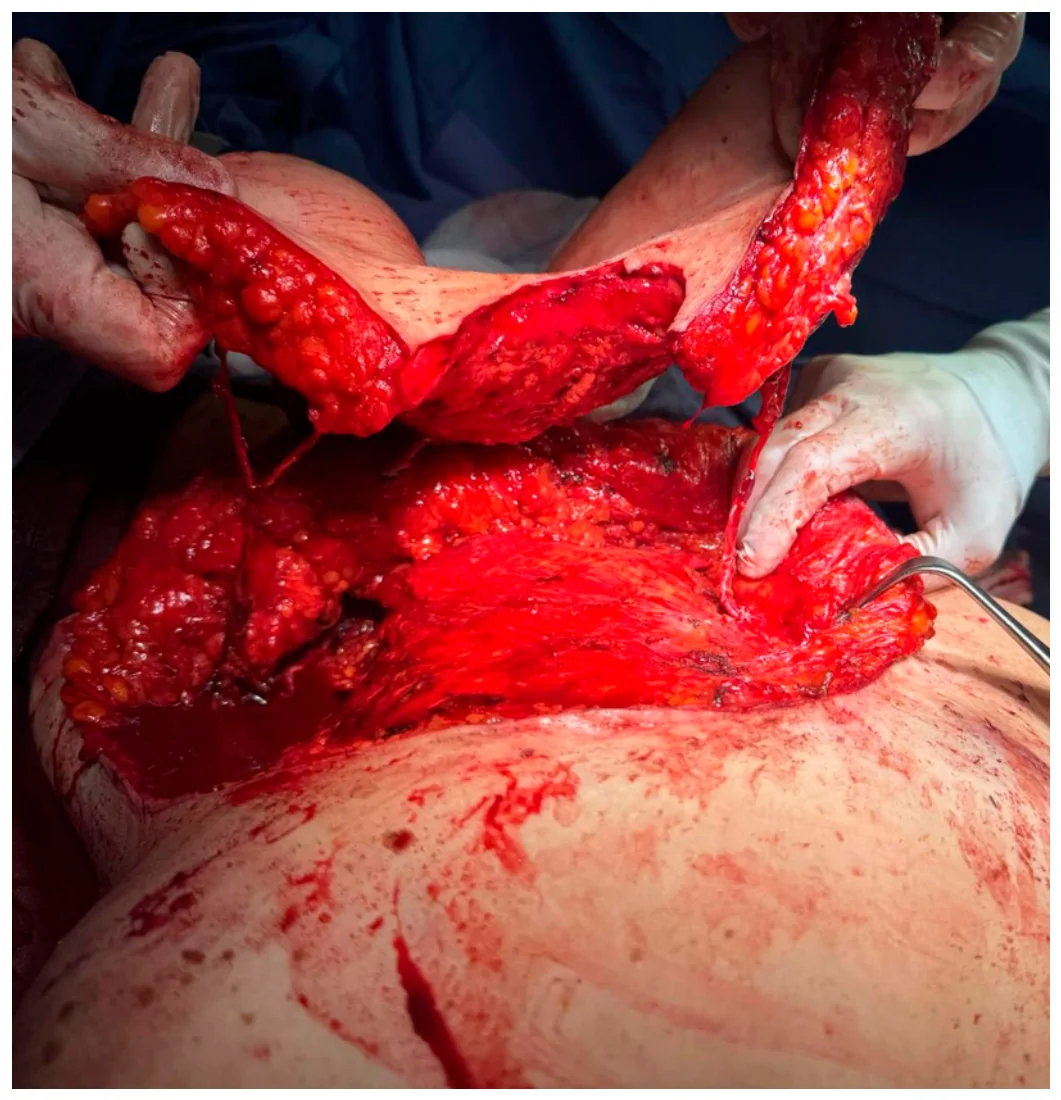
Intraoperative view of the conjoined bipedicled DIEP flap raised as a single unit, without division at the midline, and elevated on both deep inferior epigastric pedicles before pedicle division.

In the second phase, the first equipe elevates the DIEP flap on the side homolateral to the mastectomy (Figure 3), while the second equipe performs a contralateral mammary mastopexy or breast reduction for symmetry [15].

**Figure 4:**
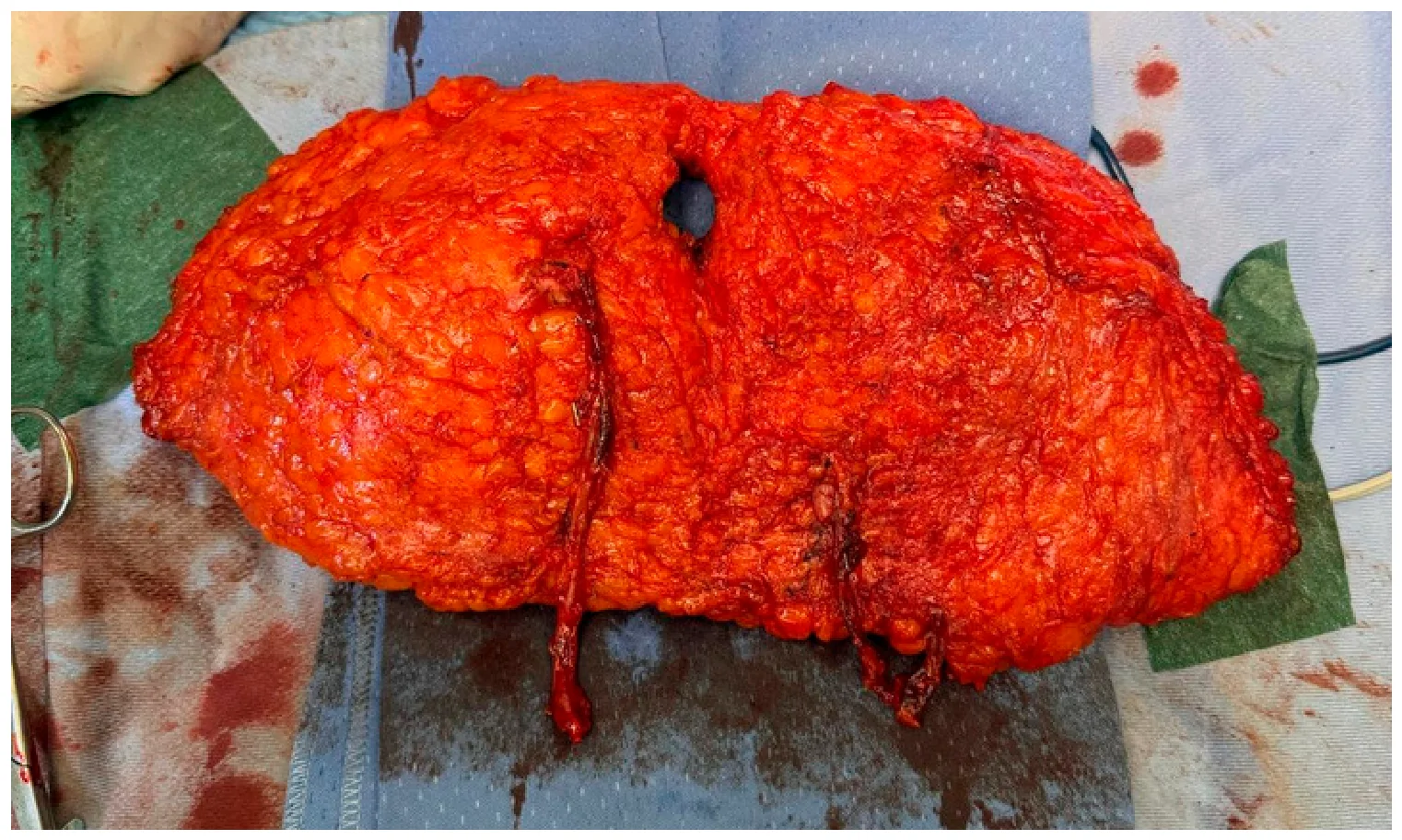
The harvested bipedicled DIEP flap (deep surface) before trimming and inset, showing the two independent vascular pedicles and the umbilical defect.

In the third phase, the first equipe trims the flap (Figure 4). Then, the same equipe performs the DIEP flap inset, while the second equipe performs abdominal closure [16,17], reinforcing the fascia with a P4HB mesh to reduce the risk of donor-site complications (routinely in bipedicled reconstructions and according to reconstructive requirement in unipedicled ones). Two drains are placed at the abdominal donor site and one in the reconstructed breast.

Microvascular anastomoses. The same anastomotic technique was used in all procedures: venous anastomoses were performed with a mechanical coupler device, whereas arterial anastomoses were hand-sewn. All internal mammary anastomoses were performed in an antegrade fashion; retrograde internal mammary vessels were not used. In bipedicled flaps, the two pedicles were anastomosed independently to two different recipient systems, one to the antegrade internal mammary vessels and the other to a branch of the axillary vessels selected as described above, so that each pedicle had its own arterial inflow and venous outflow. In unipedicled flaps, the single pedicle was anastomosed to one of the recipient systems described above.

Postoperatively, the patient is monitored every three hours for three days. Flap viability is assessed using multiple methods: vascular flow is evaluated with Doppler ultrasound, a capillary refill test is performed, flap temperature is checked, and, when necessary, dermal bleeding is assessed via needle-prick testing of the flap skin. Drains are removed when output is below 30 cc for two consecutive days, which may occur even after discharge. Following discharge, patients are seen in follow-up once or twice weekly until complete wound healing.

**Figure 5:**
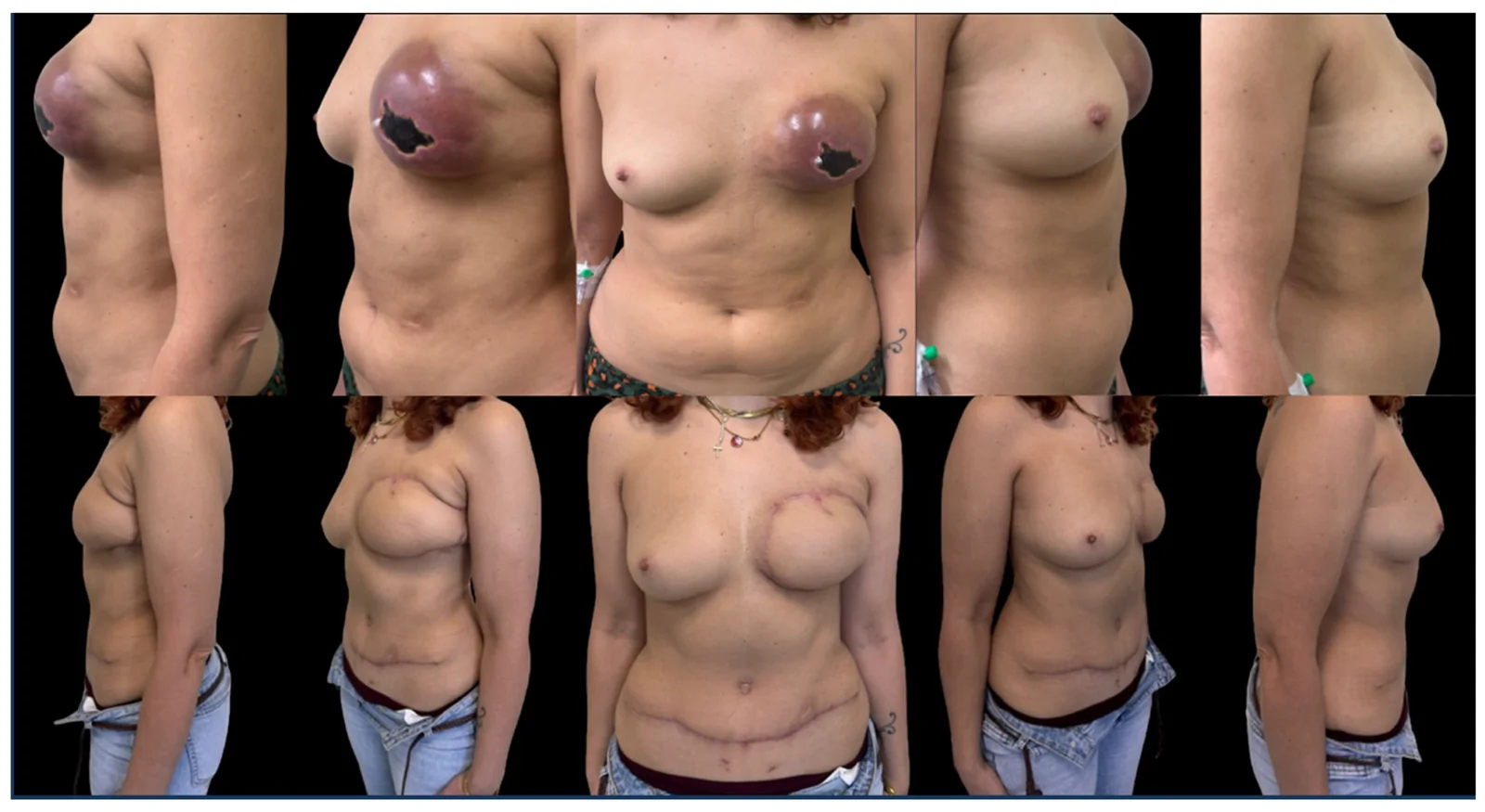
Case 1: left breast reconstruction with a bipedicled DIEP flap after a failed, previously irradiated implant-based reconstruction complicated by skin necrosis (same patient as in Figure 1). **Top** row, preoperative views; **bottom** row, postoperative views at 8-month follow-up (from left to right: lateral, oblique, frontal, oblique, and lateral views). Recipient vessels were the internal mammary and circumflex scapular vessels.

**Figure 6:**
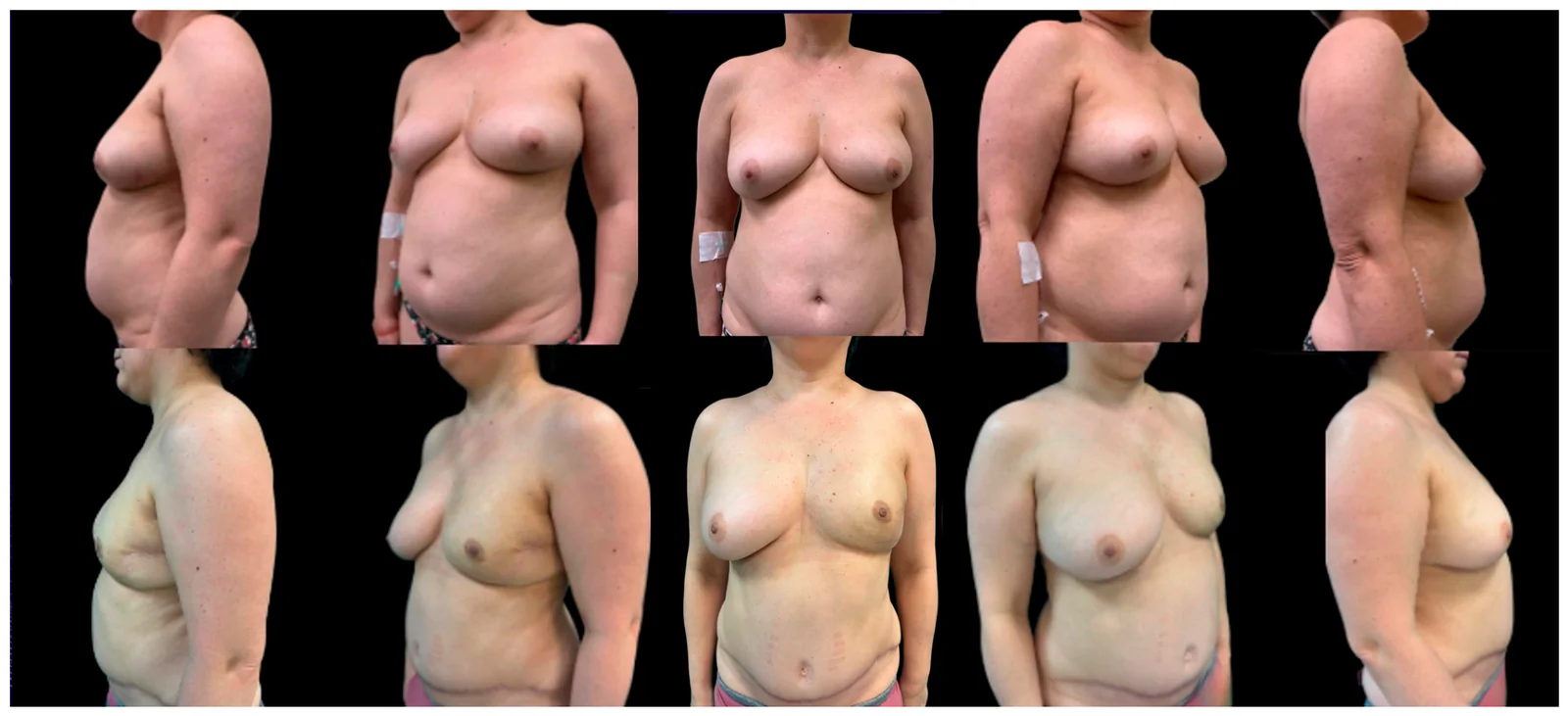
Case 2: breast reconstruction with a bipedicled DIEP flap. **Top** row, preoperative views; **bottom** row, postoperative views at 12-month follow-up (from left to right: lateral, oblique, frontal, oblique, and lateral views). Recipient vessels were the internal mammary and circumflex scapular vessels.

Representative preoperative and postoperative results of the last two patients who underwent bipedicled DIEP flap breast reconstruction are shown in Figure 5 and Figure 6 (lateral, oblique, and frontal views).

## 3. Results

### 3.1. Patient Characteristics and Operative Time

Twenty-six patients were included. Mean age was 56.5 ± 3.8 years, mean weight 69.5 ± 7.6 kg, and mean BMI 26.6 ± 3.0 kg/m^2^ (Table 1). Mean operative time was 7 h 9 min ± 47 min, longer than our usual operative time for unipedicled DIEP flap reconstruction (5 h 48 min ± 1 h 7 min; unpublished institutional data).

### 3.2. Recipient Vessels

In all 26 reconstructions, one pedicle was anastomosed to the internal mammary vessels and the other to a branch of the axillary vessels. The axillary recipient was the circumflex scapular artery in 18 cases (69.2%), the thoracodorsal artery in 7 (26.9%), and the lateral thoracic artery in 1 (3.8%).

### 3.3. Primary Outcome: Total Flap Loss

No total flap loss occurred among the 26 bipedicled flaps, compared with the approximately 4% total flap loss rate reported in the literature for DIEP flap reconstruction. These literature data, however, do not distinguish patients according to previous RT, and flap loss rates in irradiated patients may be higher (Table 2).

### 3.4. Secondary Outcomes: Flap-Related Complications

One patient (3.8%) developed a vascular complication. It was arterial and involved the pedicle anastomosed to the internal mammary vessels, and the flap survived. Skin necrosis and fat necrosis each occurred in one patient (3.8%). Minor recipient-site complications occurred in 3 patients (11.5%) (Table 2).

### 3.5. Secondary Outcomes: Donor-Site Complications

Abdominal bulge occurred in 5 patients (19.2%) and abdominal wound dehiscence in 2 patients (7.7%) (Table 2).

### 3.6. Patient-Reported Outcomes (BREAST-Q)

All 26 patients completed the BREAST-Q at the 6-month follow-up. Mean scores were 90.2 ± 12.8 for satisfaction with breasts, 91.0 ± 10.5 for satisfaction with outcome, 92.3 ± 9.8 for psychosocial well-being, 91.9 ± 12.0 for physical well-being, 91.4 ± 11.7 for sexual well-being, and 97.6 ± 4.0 for satisfaction with information (Table 3).

## 4. Discussion

Autologous breast reconstruction in the setting of radiotherapy remains one of the most challenging scenarios in microsurgical breast reconstruction. While free tissue transfer is generally considered more resilient than implant-based reconstruction in irradiated patients, radiation-induced tissue injury continues to represent a major determinant of flap-related morbidity. The chronic effects of irradiation extend beyond cutaneous fibrosis, involving progressive endothelial dysfunction, microvascular rarefaction, impaired angiogenesis, and persistent inflammation, ultimately resulting in a recipient bed characterized by reduced vascular reserve and compromised healing capacity [18,19]. Even when microsurgical anastomoses remain patent, reconstructive outcomes continue to be influenced by the hostile biological environment created by radiotherapy, where impaired tissue perfusion, altered microcirculation, and reduced regenerative capacity limit flap integration and wound healing [20,21,22].

Against this background, the present study suggests that bipedicled DIEP flap reconstruction may represent a valuable strategy to improve reconstructive reliability in irradiated patients [23]. The most relevant finding was the complete absence of total flap loss in the bipedicled cohort compared with a 4% flap loss rate described in litterautre for unipedicled reconstruction. Although statistical significance was not reached for secondary outcomes, all clinically relevant flap-related complications, including vascular compromise, fat necrosis, skin necrosis, and delayed wound healing, occurred less frequently after bipedicled reconstruction. While these findings should be interpreted considering the limited sample size, the consistency of the observed trends raises the hypothesis that the potential benefit of pedicle duplication may extend beyond flap survival alone [24].

The rate of total flap loss observed in the unipedicled group deserves specific consideration, as it exceeds the rates generally reported for DIEP flap reconstruction, which are commonly below 4%. However, the effect of radiotherapy on total flap loss remains controversial. A recent meta-analysis of DIEP flap reconstructions found higher rates of partial flap loss and wound revisions but no significant difference in total flap loss in irradiated breasts [25], whereas other studies have suggested that radiotherapy may increase the risk of microvascular and thrombotic complications in free tissue transfer through pathophysiological mechanisms that have not been fully elucidated [18,20,21]. Because total flap loss is a rare event, most available series lack the statistical power to detect a difference in this specific subgroup, and pooled rates may therefore not fully reflect the risk in irradiated recipient beds. In our institution, the flap loss rate after DIEP reconstruction in non-irradiated patients is according to the present literature about DIEP reconstruction (unpublished institutional data), which argues against a purely technique-related explanation of the present findings; nevertheless, this comparison was not part of the study design and should not be regarded as conclusive. Moreover, whereas vascular complications of DIEP flaps are typically venous, in our experience vascular complications in irradiated patients were more frequently arterial, which is in keeping with the endothelial and intimal injury induced by radiotherapy in recipient vessels [18,20].

Traditionally, the indication for bipedicled DIEP reconstruction has been driven primarily by anatomical considerations, including large flap volume requirements, limited hemi-abdominal tissue availability, or concerns regarding perfusion across the midline [25,26]. Our findings are consistent with the hypothesis that the condition of the recipient bed should also be considered when selecting flap configuration. In patients previously exposed to radiotherapy, where tissue vascularity and regenerative capacity are intrinsically compromised, the additional vascular redundancy provided by a second pedicle may enhance flap resilience and reduce the risk of ischemia-related complications. In our series, these potential benefits were not accompanied by a detectable increase in early donor-site morbidity despite the greater technical complexity of bipedicled flap harvest, a finding that is consistent with previous comparative studies evaluating uni- and bipedicled DIEP reconstruction [27,28].

The potential advantages of a bipedicled DIEP configuration are also supported by the unique hemodynamic characteristics of perforator flaps. Experimental studies by Rubino et al. demonstrated that elevation of a perforator flap profoundly modifies blood-flow dynamics, giving rise to the so-called inversion phenomenon, in which blood flow becomes preferentially directed toward the perforator circulation with a marked increase in flow velocity compared with the native vascular state [29]. These findings challenged the traditional concept of flap perfusion by showing that vascular physiology is substantially reorganized following perforator flap harvest rather than simply preserved. Subsequent studies further demonstrated that these hemodynamic changes are accompanied by adaptive modifications of the flap microcirculation, with increased blood velocity within the perforator system and progressive optimization of tissue perfusion. Moreover, flap perfusion has been shown to depend on a dynamic relationship between vascular flow and the perfused tissue territory, emphasizing that arterial inflow and venous drainage are critical and that increasing the drainage veins protects against the possibility of flap venous failure.

Collectively, these observations suggest that perforator flaps should be regarded as dynamic vascular networks rather than static anatomical territories. In our bipedicled DIEP technique, the flap remains a single continuous tissue unit supplied and drained by two independent vascular systems. Unlike reconstructions based on two separate flaps, pedicle duplication does not divide the flap into two separate perfusion territories but introduces a second source of arterial inflow and venous drainage into the same interconnected microvascular network.

From a systems perspective, pedicle duplication may increase the functional redundancy of the reconstructed vascular network. Rather than relying on a single arterial inflow and venous outflow axis, the flap is supported by two independent vascular systems capable of contributing to the perfusion of a single interconnected microvascular network. Although the extent of this functional redundancy remains to be elucidated, such an architecture may increase the resilience of the reconstructed circulation to localized vascular disturbances occurring during the early postoperative period.

We therefore hypothesize that the principal advantage of pedicle duplication lies not in increasing absolute blood flow but in modifying the hemodynamic architecture of the reconstructed vascular network. The presence of two independent inflow–outflow systems may increase vascular redundancy and improve the ability of the flap circulation to redistribute perfusion when transient postoperative perturbations—such as vasospasm, tissue edema, or temporary venous impairment—occur. This adaptive capacity may be particularly important in irradiated recipient beds, where endothelial dysfunction, fibrosis, and microvascular rarefaction reduce the physiological ability of the tissue to compensate for hemodynamic stress. Although this mechanism has not been directly demonstrated experimentally, it provides a physiologically coherent interpretation of the lower incidence of flap-related complications observed in our cohort.

It should be emphasized that the purpose of pedicle duplication is not to prevent anastomotic thrombosis per se. It is plausible that the incidence of anastomotic failure was similar in the two groups and that the difference in flap survival reflects the presence, in bipedicled flaps, of a second pedicle acting as a vascular backup: because each perforator also perfuses, through choke vessels, the territory of the contralateral perforator, thrombosis of one pedicle does not necessarily result in flap loss. Whether both pedicles remained patent throughout follow-up was not investigated in the present study, and this interpretation therefore requires confirmation.

Current evidence has consistently supported the use of bipedicled DIEP flaps as a reliable reconstructive option when donor-site anatomy limits the feasibility of a conventional unipedicled reconstruction. Historically, pedicle duplication has been adopted to increase flap volume, improve perfusion across the midline, or overcome unfavorable perforator anatomy, particularly in unilateral large-volume breast reconstruction [30]. More recently, comparative studies and systematic reviews have confirmed that bipedicled DIEP reconstruction is a safe and reproducible technique, with donor-site morbidity largely comparable to conventional unipedicled DIEP reconstruction despite its greater technical complexity. Although individual series have reported slightly higher rates of donor-site complications following bipedicled harvest, these differences appear to be primarily related to longer operative times rather than pedicle duplication itself. These findings have progressively shifted the perception of bipedicled reconstruction from a rescue procedure to a reproducible microsurgical strategy in selected patients [31].

However, the criteria guiding patient selection have remained predominantly donor-oriented, focusing on flap volume requirements and vascular anatomy rather than on the biological characteristics of the recipient bed. Although recent reports have successfully applied bipedicled DIEP reconstruction in increasingly challenging clinical settings—including previous irradiation, extensive abdominal scarring, and compromised vascular conditions—the rationale for pedicle duplication has largely remained technical, aiming to maximize vascular safety in complex reconstructions rather than addressing the biological consequences of recipient-bed injury [32].

Building upon this experience, our findings support a complementary perspective. Rather than considering previous radiotherapy solely as a technical challenge associated with higher reconstructive complexity, we propose that it should be regarded as a defining biological characteristic of the recipient bed that may itself influence flap configuration. Within this framework, pedicle duplication is not intended simply to increase flap volume or expand the perfused territory but to improve the ability of the reconstructed vascular system to function within a biologically compromised environment. Accordingly, recipient-bed physiology should be considered alongside donor-site anatomy, perforator characteristics, and flap volume requirements as an additional determinant of pedicle selection during preoperative planning.

A potential concern associated with bipedicled DIEP reconstruction is the greater operative complexity resulting from bilateral pedicle dissection, which inevitably prolongs operative time and may theoretically increase donor-site morbidity. However, comparative studies have consistently shown that donor-site complications remain largely comparable with those observed after conventional unipedicled DIEP reconstruction, with operative duration rather than pedicle duplication itself appearing to be the principal determinant of postoperative abdominal morbidity. Nevertheless, bipedicled harvest has been associated in the literature with a higher risk of abdominal bulging [10,11]. In our series, abdominal bulge rates were comparable between groups, which we attribute at least in part to the systematic fascial reinforcement with a P4HB mesh; however, the 6-month minimum follow-up is insufficient to exclude late-onset bulges or hernias, which may become apparent later and may be more frequent after bipedicled harvest. The overall increase in our operative time is approximately 1 h and 20 min. This is mainly achieved by dividing the operation into coordinated phases and allowing the two teams to work in parallel. The additional operative time should therefore be considered in relation to the potential benefits of the technique: more balanced flap perfusion, safer use of an adequate tissue volume, removal of the least reliably perfused Hartrampf zones, and the presence of a second vascular pedicle that may support the flap if the other pedicle becomes compromised.

An additional consideration is the educational value of bipedicled DIEP flap reconstruction for microsurgical training. Compared with a conventional unipedicled DIEP flap, each bipedicled procedure requires approximately twice the number of microvascular anastomoses, thereby substantially increasing the technical workload within a single operation. This greater procedural complexity exposes the surgeon to a higher volume of microsurgical tasks over the same operative period, potentially accelerating the development of technical proficiency and shortening the microsurgical learning curve. Beyond the refinement of anastomotic technique, repeated execution of multiple vascular anastomoses may improve operative efficiency, tissue handling, intraoperative decision-making, and the ability to manage anatomical variability and unexpected technical challenges. Consequently, although bipedicled DIEP flap reconstruction is a generally demanding procedure in autologous breast reconstruction, its adoption may provide a disproportionate educational benefit for surgeons developing advanced microsurgical expertise, provided that appropriate patient selection, supervision, and institutional experience are ensured.

### 4.1. Clinical Implications

The present findings may have implications for surgical decision-making in autologous breast reconstruction following radiotherapy. Rather than representing an isolated change in practice, the present study provides preliminary quantitative data supporting an evolution in reconstructive strategy that had progressively emerged from our institutional experience. Based on these observations, previous chest-wall radiotherapy is now considered an indication for bipedicled DIEP reconstruction within our institutional algorithm, irrespective of conventional donor-site criteria such as flap volume or perforator anatomy.

We believe that this approach reflects a broader conceptual shift in flap selection. While donor-site anatomy remains fundamental for operative planning, the biological condition of the recipient bed should also influence pedicle configuration. In our experience, incorporating recipient-bed vascular physiology into the decision-making process appears to have improved the reliability of autologous reconstruction in irradiated patients. Future prospective and multicenter studies will be required to determine whether this strategy can be generalized beyond our institutional practice.

### 4.2. Limitations

The present study has several limitations. First, its retrospective design and the relatively small sample size limit the statistical power to detect differences in secondary outcomes and may introduce selection bias. Second, no control group of non-irradiated patients was included; therefore, it cannot be established whether the benefit of pedicle duplication is specific to irradiated recipient beds or would also be observed in non-irradiated patients, and factors other than radiotherapy may have contributed to the observed outcomes. Third, the minimum follow-up of 6 months is adequate for flap-related outcomes but could limit the reliability of the analysis of late donor-site complications such as abdominal bulging and hernia. Fourth, the patency of both pedicles in bipedicled flaps was not assessed during follow-up; it therefore remains unknown whether both anastomoses remained functional or whether flap survival was sustained by a single pedicle in some cases. Finally, the proposed hemodynamic model remains hypothetical, as no quantitative assessment of flap perfusion, intraoperative flow dynamics, or postoperative microvascular adaptation was performed. Consequently, the physiological mechanisms underlying the observed reduction in flap-related complications should be considered hypothesis-generating and warrant prospective investigation.

## 5. Conclusions

In conclusion, bipedicled DIEP reconstruction was associated with improved flap survival in previously irradiated patients, without an apparent increase in early donor-site morbidity. Beyond its established anatomical indications, our findings raise the hypothesis that recipient-bed vascular physiology may also be relevant to flap selection when planning autologous breast reconstruction after radiotherapy. Prospective multicenter studies including non-irradiated controls, longer follow-up, and quantitative perfusion assessment will be essential to validate this concept and determine its broader applicability.

## Figures and Tables

**Table 1:** Patient characteristics and operative time (n = 26).

Characteristic	Bipedicled (n = 26)
Age, years	56.5 ± 3.8
Weight, kg	69.5 ± 7.6
BMI, kg/m^2^	26.6 ± 3.0
Operative time, h:min	7:09 ± 0:47

Values are expressed as mean ± standard deviation. BMI, body mass index.

**Table 2:** Flap-related and donor-site outcomes (n = 26).

Outcome	Bipedicled (n = 26)
Primary outcome	
Total flap loss	0 (0)
Secondary outcomes: recipient site	
Vascular complications	1 (3.8)
Skin necrosis	1 (3.8)
Fat necrosis	1 (3.8)
Minor complications	3 (11.5)
Secondary outcomes: donor site	
Abdominal bulge	5 (19.2)
Wound dehiscence	2 (7.7)

Values are expressed as the number of patients (%). Minor complications: composite of seroma, hematoma, infection, and wound dehiscence with delayed healing at the recipient site.

**Table 3:** BREAST-Q scores at 6-month follow-up (n = 26).

Domain	Mean ± SD	Range
Satisfaction with breasts	90.2 ± 12.8	45–100
Satisfaction with outcome	91.0 ± 10.5	55–100
Psychosocial well-being	92.3 ± 9.8	60–100
Physical well-being	91.9 ± 12.0	43–100
Sexual well-being	91.4 ± 11.7	60–100
Satisfaction with information	97.6 ± 4.0	85–100

Scores range from 0 to 100, with higher scores indicating better outcomes; values are expressed as mean ± standard deviation and range (minimum–maximum).

## Data Availability

The data presented in this study are available in the article.
